# An intuitive surgical handle design for robotic neurosurgery

**DOI:** 10.1007/s11548-021-02402-4

**Published:** 2021-05-24

**Authors:** Emmanouil Dimitrakakis, Lukas Lindenroth, George Dwyer, Holly Aylmore, Neil L. Dorward, Hani J. Marcus, Danail Stoyanov

**Affiliations:** 1 Wellcome/EPSRC Centre for Surgical and Interventional Sciences (WEISS), University College London (UCL), London, UK; 2 UCL Queen Square Institute of Neurology, London, UK; 3 National Hospital for Neurology and Neurosurgery, London, UK

**Keywords:** Medical robotics, Neurosurgery, Robotic-assisted endonasal approach, Handheld robotics, Ergonomics

## Abstract

****Purpose**:**

The expanded endoscopic endonasal approach, a representative example of keyhole brain surgery, allows access to the pituitary gland and surrounding areas through the nasal and sphenoid cavities. Manipulating rigid instruments through these constrained spaces makes this approach technically challenging, and thus, a handheld robotic instrument could expand the surgeon’s capabilities. In this study, we present an intuitive handle prototype for such a robotic instrument.

****Methods**:**

We have designed and fabricated a surgical instrument handle prototype that maps the surgeon’s wrist directly to the robot joints. To alleviate the surgeon’s wrist of any excessive strain and fatigue, the tool is mounted on the surgeon’s forearm, making it parallel with the instrument’s shaft. To evaluate the handle’s performance and limitations, we constructed a surgical task simulator and compared our novel handle with a standard neurosurgical tool, with the tasks being performed by a consultant neurosurgeon.

****Results**:**

While using the proposed handle, the surgeon’s average success rate was $80\%$, compared to $41\%$ when using a conventional tool. Additionally, the surgeon’s body posture while using the suggested prototype was deemed acceptable by the Rapid Upper Limb Assessment ergonomic survey, while early results indicate the absence of a learning curve.

****Conclusions**:**

Based on these preliminary results, the proposed handle prototype could offer an improvement over current neurosurgical tools and procedural ergonomics. By redirecting forces applied during the procedure to the forearm of the surgeon, and allowing for intuitive surgeon wrist to robot-joints movement mapping without compromising the robotic end effector’s expanded workspace, we believe that this handle could prove a substantial step toward improved neurosurgical instrumentation.

## Introduction

Robotic-assisted minimally invasive surgery (RAMIS), with its precise instrument articulation and dexterity [25], could be important in the future of a variety of surgical operations and especially in the future of neurosurgery. Wristed instruments, augmented reality, and stereoendoscopy could have a significant impact in these complex procedures [22]. A specific neurosurgical operation that could greatly benefit from robotic articulation is the expanded endoscopic endonasal approach (EEEA), which is a minimally invasive surgery (MIS) technique that is performed through the natural orifice of the nose and aims at the removal of sellar and parasellar lesions [8].

Although a promising alternative to transcranial approaches that require craniotomies and brain retraction, the EEEA comes with its limitations. In [20], $74\%$ of the surgeons surveyed identified the limited surgical manipulation that the standard non-articulated instruments offer as the biggest challenge. A handheld surgical robotic instrument could potentially provide this missing manipulation and expand the neurosurgeon’s capabilities.

The purpose of this study is to prototype and validate an ergonomic and intuitive handle for such a robotic instrument. The handle’s novel intuitive design consists of a 4-degree-of-freedom (DoF) joystick that directly maps the surgeon’s wrist to the robotic end-effector joints so that it offers a fast adoption rate. This added robotic dexterity could potentially improve the surgery outcome and patient health by increasing the surgeon’s operative workspace. Other than increasing the surgeon’s capabilities, we also aim to alleviate their wrist of any added load and fatigue by mounting the handle on the surgeon’s forearm and thus make the procedure more ergonomic and comfortable. To evaluate the performance and limitations of this handle, a comparative experiment between the prototype and a standard rigid neurosurgical tool was carried out in a custom-built surgical task simulator with physical constraints that resembled the limited space of the EEEA.

## Related work

The field of research on handheld surgical robots is densely populated with a number of promising studies. In [26], a mechatronic hand-held surgical instrument is developed with the goal of achieving a trade-off between dexterity, intuitiveness of control and weight of the system. A handheld robotic platform for natural orifice transluminal endoscopic surgery (NOTES) was developed in [32], with the initial in vivo animal trials demonstrating the robot is straightforward to use. Complimentary to these studies is the study in [16], where a novel handheld mechatronic instrument was presented and tested both in vitro and in vivo, accompanied by an intuitive and ergonomic interface. Finally, another study that focused on the ergonomic design of the handle, as well as the implementation of a modular dexterous handheld surgical robot, is that in [35].

There are also a number of studies that inspect the efficacy of robotized surgical tools, both commercial and non-commercial in simulated or realistic clinical scenarios or by comparing them to conventional tools. In [14], the Kymerax system was evaluated during a laparoscopic hysterectomy and showcased potential for laparoscopy. In [4], it is suggested that the robotized needle holder Jaimy2, when compared with a conventional needle holder, improves both the surgeon’s posture and the quality of laparoscopic sutures. Another study that compared a handheld robot with a conventional tool is [30], where a motor-driven laparoscopic needle holder resulted in similar suturing performance with that of the conventional tool, but better ergonomics of the surgeon’s hand posture.

It is worth mentioning that developing robotic systems is not the only solution explored to increase the dexterity and articulation of current surgical tools. Such notable non-robotic attempts can be found in [3, 15], where the purely mechanical surgical tools are attached to the surgeon’s forearm, as is the case in this study. While non-robotic systems such as these can prove very beneficial to current surgical operations and can provide the surgeon with direct force feedback, they lack the variable motion scaling and the robotized guidance that surgical robotic instruments could offer.

**Table 1:** Design criteria for the proposed intuitive handle prototype

The same handle design should cater to different hand sizes.
The handle actuation means, namely the trigger, should be finger-operated.
A resting hand pose should hold the instrument at rest.
The wrist should not support much weight throughout the duration of the procedure.
The surgeon’s wrist motions should be mapped to the robot-joints motions.
The handle should provide a platform to incorporate more complex robotic assistance.

When it comes to the EEEA procedure, most proposed robotic systems are tele-operated, such as the one in [2], where the end effector of the robot is articulated with deformable parts, and in [5], which employ concentric tube robots. Even though these systems could have an impact in the operating theatre, handheld robots are usually more compact, traditionally have smaller purchasing and maintenance costs, and their smaller footprint makes their integration into the surgical workflow easier [24].

## Methods

### Design criteria

Long-term use of non-ergonomically designed tools can cause conditions such as carpal-tunnel syndrome [33] and other significant ergonomic problems, such as pain in the regions of the neck and upper extremities [17]. Thus, appropriate ergonomic tool design is essential. The goal of our study is to develop a safe and effective handle that focuses on ergonomics and comfort and that has a fast adoption rate, so that it can be easily integrated in the operating theatre.

According to [10], it is difficult to define a universal consensus on specific guidelines that make a handle design ergonomic and comfortable to use. Despite this difficulty, there are still some characteristics that are found to be contributing toward an ergonomic design. In [13], it is found that the size of the surgeon’s hand is related to the handle size that they consider optimal, and in [36], it is proposed that the preferable handle manipulation type is finger-operated, specifically with the thumb and index finger. Additionally, in [28] it is suggested that in order for a handle to feel comfortable, when the instrument is kept at rest, the surgeon’s hand should maintain a partially opened pose.

Handle weight has a significant effect on muscle load when performing manual tasks [12]. Transferring the weight that the wrist supports to the forearm could ease the wrist load and reduce fatigue. Simultaneously, with this setup, the end effector is decoupled from the wrist, meaning that any forces applied on the distal end of the instrument, that would normally be directly transferred back to the wrist, are now directed to the forearm. Mounting the instrument on the surgeon’s forearm, creates the possibility to map the surgeon’s wrist directly to the robot joints which could increase the chance of developing a surgical instrument with good usability [15] and could possibly ease the realization of difficult tasks such as suturing, by replicating the full range of motion of the surgeon’s hand.

All aforementioned instructions and suggestions lead to the following design criteria defined for our handle summarized in Table 1.

### Handle design

**Fig. 1:**
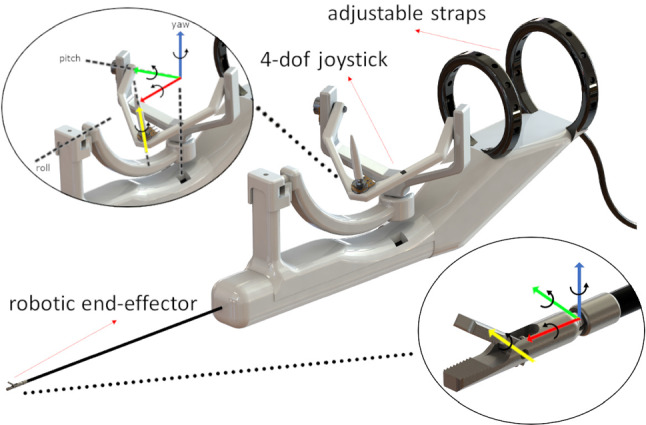
A rendering of the suggested intuitive handle with the coordinate frames of the handle joints (left) and the corresponding coordinate frames of the robot joints (right)

A rendering of the suggested intuitive handle, that was designed trying to adhere to the design criteria set in “Design criteria” section, is found in Fig. 1.

**Fig. 2:**
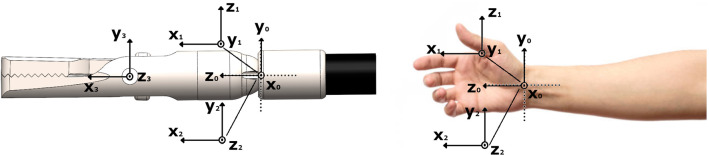
The coordinate frames of the end effector that the handle is intended to manipulate, and the corresponding frames on the wrist

The robotic end effector that this handle is intended to manipulate is a tendon-driven three-DoF robot, with a diameter of 3.6*mm* and 1.97*cm* length, and has been developed in previous work [11]. It consists of a single spherical joint that provides the yaw and pitch DoF, with the third DoF being the opening and closing of the gripper. The coordinate frames in 3D space of the end effector are shown in Fig. 2. In the same figure, an added frame for the roll motion is evident, which is carried out by the motorized rotation of the outermost part of the handle body that is connected with the end-effector shaft.

The handle itself is larger than currently used tools but lightweight, with maximum dimensions of 42*cm* length, 16*cm* height, and a weight of 431*gr* in its current iteration. It consists of a 4-DoF joystick that resembles three axes of rotation gimbal structure, with an additional DoF for the gripper actuation. This gripper DoF is finger-operated, namely with the index finger and the thumb, with a rolling pinch motion. The surgeon’s hand rests at the base of the joystick, and the three axes of rotation, disregarding the gripper actuation axis, form an imaginary origin in space that coincides with the surgeon’s wrist. The angle of each handle joint is measured by a potentiometer that controls each end-effector joint in joint space.

**Fig. 3:**
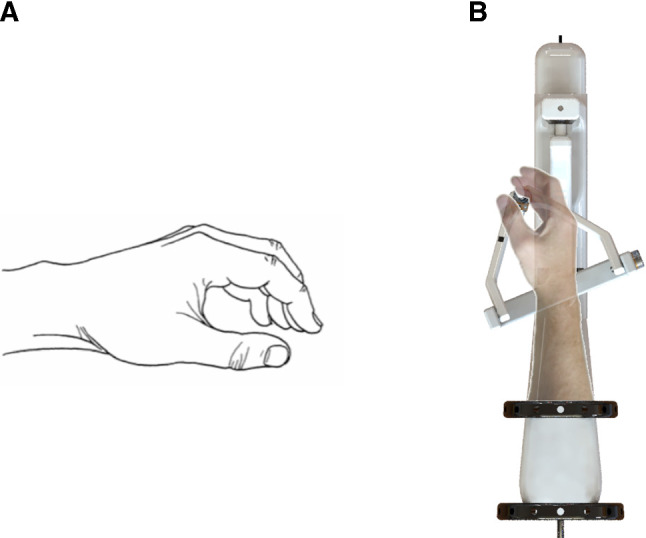
**a** The hand at its resting position [19] and **b** the resting position of the handle coincides with the resting pose of the wrist

Looking back at the design criteria that were set in “Design criteria” section, our suggested handle is finger-operated in part, and its three axes of rotation immediately map the surgeon’s wrist yaw, pitch and roll rotations. At a handle resting position, namely when the surgeon is not manipulating the robot joints, the surgeon’s hand is also maintained at a resting pose, as evident in Fig. 3.

The handle is forearm-mounted with adjustable straps, meaning that all weight is alleviated from the wrist and transferred to the forearm. Since a mounting point, such as a trocar, is generally missing during the EEEA procedure, the adjustable straps constrain the handle to the surgeon’s forearm creating a stable platform on which the surgeon can freely move their wrist. The base frame of the end effector is directly manipulated by the surgeon’s arm, as the instrument shaft is rigidly fixed parallel to the surgeon’s forearm.

### Prototype fabrication

**Fig. 4:**
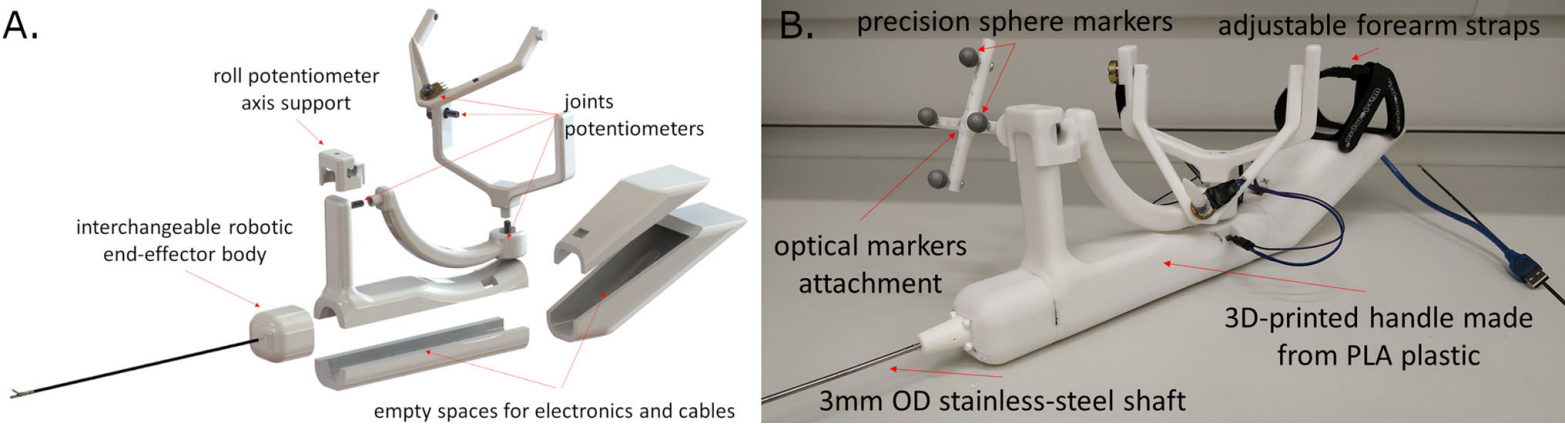
**a** Exploded view of the proposed handle rendering, and **b** its 3D-printed implementation (right)

The handle prototype was implemented with additive manufacturing techniques, namely 3D printing. All parts of the handle were 3D-printed (Ultimaker S5, Ultimaker BV, Utrecht, Netherlands), using polylactic acid (PLA). For the end-effector shaft, a $3-mm$-diameter stainless steel rod was used. The joint angle values were measured with 4 rotary potentiometers and were read and handled with the use of a miniature single-board microcontroller (Arduino Nano, Arduino AG, Italy). The inside of the handle body contains only the microcontroller, a mini-bread board to connect it with the potentiometers and the cables. To mount the handle to the forearm, 2 12-inch hook-and-loop adjustable straps were used. An exploded view of the handle with more details on the design, alongside the 3D printed prototype with its optical marker, is shown in Fig. 4.

## Experimental methods

To evaluate the performance of the handle, and more specifically its intuitiveness, and whether mounting it on the surgeon’s forearm would reduce the benefits of the added articulation and ergonomic design as [1] suggests, we built a custom surgical task simulator to compare it with a standard rigid instrument that is commonly used in keyhole brain surgery. The task we chose to simulate was a ‘peg-transfer’ task, taken from the McGill Inanimate System for Training and Evaluation of Laparoscopic Skills (MISTELS) [9]. This task was also evaluated in [21], as indicative of surgical skill when carried out in constrained spaces, such as those of keyhole brain surgery. The focal investigative point of our experiment is whether our proposed handle can still outperform the conventional tool, while alleviating the wrist of any added fatigue, something that would indicate that there is no loss of robotic dexterity.

To investigate whether that is the case, our simulation platform and specifically the peg-board dimensions have been designed in accordance with specifications from [21] that describe an experimental setup that would correspond to the constrained operative workspace of keyhole brain surgery. Some of the pegs were purposefully positioned in coordinates where it would be difficult for the standard instrument to reach them, to highlight the importance of articulation, and evaluate whether the forearm mount limits the dexterity of the end effector.

### Surgical task simulator

The conventional tool that was used for the study was a 28164TA surgical forceps (Karl Storz SE & Co. KG), on which a push button and a microcontroller were placed to simulate the grasping motion. Additionally, a 3D-printed endoscopic device was fabricated to manipulate the camera and its field of view, throughout the duration of the task. The manufactured device was inspired by the $4-mm$ Endocameleon Neuro Hopkins Endoscope (Karl Storz SE & Co. KG).

**Fig. 5:**
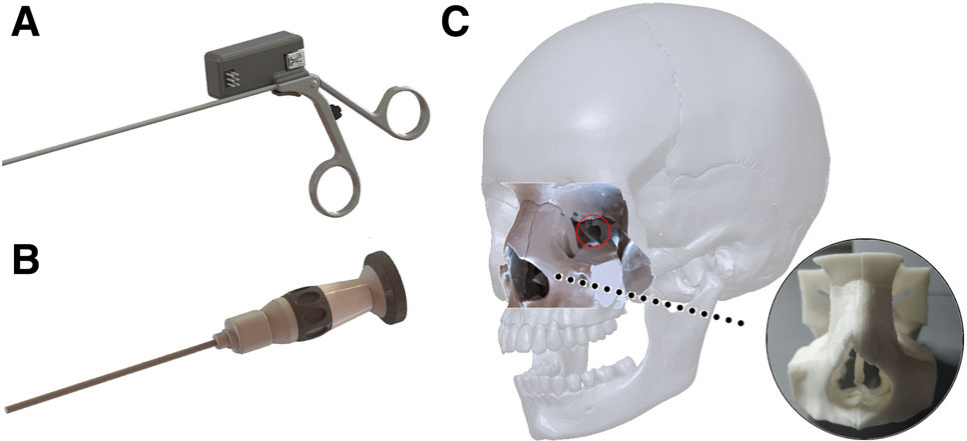
**a** Rendering of the modified surgical forceps, **b** rendering of the endoscope and **c** rendering of the physical nasal channel constraint, with the artificial channel circled, a whole skull for reference, and the 3D-printed phantom constraint

To try and realistically replicate the physical constraint of the EEEA, a 3D model of a cranial CT scan was also prototyped, modified so that it only features the EEEA areas of interest. This phantom constraint also guides the endoscope to be placed at the angle and distance from the pituitary gland area that the endoscope would be placed in a real surgical operation, providing a similar camera field of view and video feed to that of the procedure. This physical phantom constraint included an $1-cm$-diameter artificial cylindrical channel passing through the sphenoid sinus and granting access to the pituitary gland area. This channel is drilled-through by the surgeon during the actual surgery [6]. The modified conventional tool, the endoscope and the phantom are shown in Fig. 5.

The simulation environment used to develop the ‘peg-transfer’ task was the CoppeliaSim (formerly V-REP) [29] simulation platform. To simulate the conventional tool, the same CAD model as the robotic end effector was used, but without the robotic joints, to avoid differences in the surgeon’s performance due to difference in tools geometries. Communication between the tools and the simulation environment was achieved through the robot operating system (ROS) [27]. All prototyped tools, as well as the constraint, were optically tracked using the motion capture system (Optitrack V120:trio, NaturalPoint Inc., Canada) and custom marker attachments. Each rigid body was tracked with 4 $11-mm$ spherical markers. The attachment with all four markers attached to it weighed less than 15*gr*, deeming the marker setup weight negligible when compared to the overall device weight. Thus, we can safely assume that it did not affect the ergonomics of the device.

The peg base was placed in 3D space at specific offsets from the constraint markers and the tracking for both instruments was calibrated in such a way so that when the shaft of each instrument would touch the desk, that point would correspond to a point just below the peg-board in the 3D space of the simulation, so as to not obstruct the surgeon’s motion. This simple solution aimed to give the user subject of the study a hard limit when inserting the instrument’s shaft inside the physical phantom constraint and thus a basic feel for haptics.

**Fig. 6:**
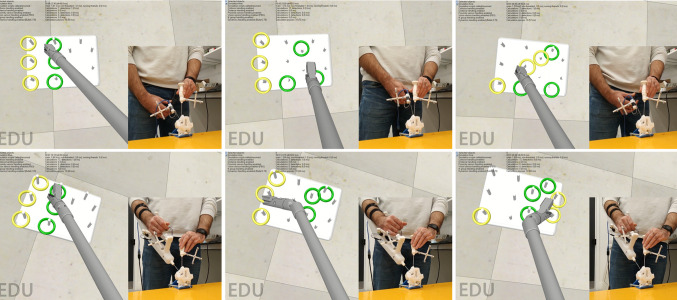
The custom-built simulated surgical task using the conventional tool (upper row) and the proposed handle (bottom row)

The simulation environment, the instruments, constraint and their markers are shown in Fig. 6. The constraint markers are not shown, because they were placed at a position where they would not block the view of the tool markers.

As previously mentioned, the purpose of this custom simulation environment was to design a comparative experiment to assess the intuitiveness and shortcomings of the proposed handle. To do this, it was necessary to evaluate the behavior of the handles kinematically, rather than to offer a hyper-realistic simulation environment. This leads to some minor inaccuracies in object interaction, such as mesh clashing, which, however, did not affect the efficacy of the experiment. Since there is no intention for this simulator to be used for training or other demonstration purposes and since the simulation parameters are the exact same for both tools as to not give advantage to one over the other, we decided that hyper-realism in interactions should not be a focal point in development.

### Comparative experiment

An expert neurosurgeon specializing in EEEA procedures was tasked with running the comparative experiment to help evaluate the efficacy of the handle prototype. An initial single minute test run for each tool was carried out so that the surgeon familiarizes themselves with the simulation environment and handling of the tools. Upon completion of the test runs, a total of 20 attempts to transfer all hoops from the set of pegs on the left to that on the right were carried out for each tool, with a maximum duration of 2 minutes each. The surgeon carried out all 20 repetitions of the task using the conventional tool, before switching to the proposed handle.

The objective metrics used to compare the two instruments were the number of attempts it took to complete the task for the first time, and the task success rate. Additionally, the learning curve when operating with the handles was investigated. To examine the ergonomics of the proposed handle, a researcher was observing the surgeon during the experiment completing parts of the Rapid Upper Limb Assessment (RULA) [23], a validated measure to assess the ergonomics of operating posture. Finally, after the experiment was completed, the surgeon was asked to complete the Surgery Task Load Index (SURG-TLX) questionnaire [34] for each of the two tools, the most commonly used subjective assessment of perceived cognitive load among individuals within a surgical team.

## Results

The number of tries until first completion, the average success rate and the successful attempts rate are summarized in Table 2. Here, the success rate is defined as the rate of hoops successfully transferred from one set of pegs to the other, over the total amount of hoops, whereas the successful attempt rate is defined as the rate of completed tasks, with all 6 hoops transferred from one set of pegs to the other, over the total number of tasks.

**Table 2:** Comparative table between the conventional tool and the proposed handle

Tool	Conventional tool	Proposed handle
Attempts until first completion	NA	2
Average success rate	$40.83\%$	$80\%$
Successful attempts rate	$0\%$	$20\%$

**Fig. 7:**
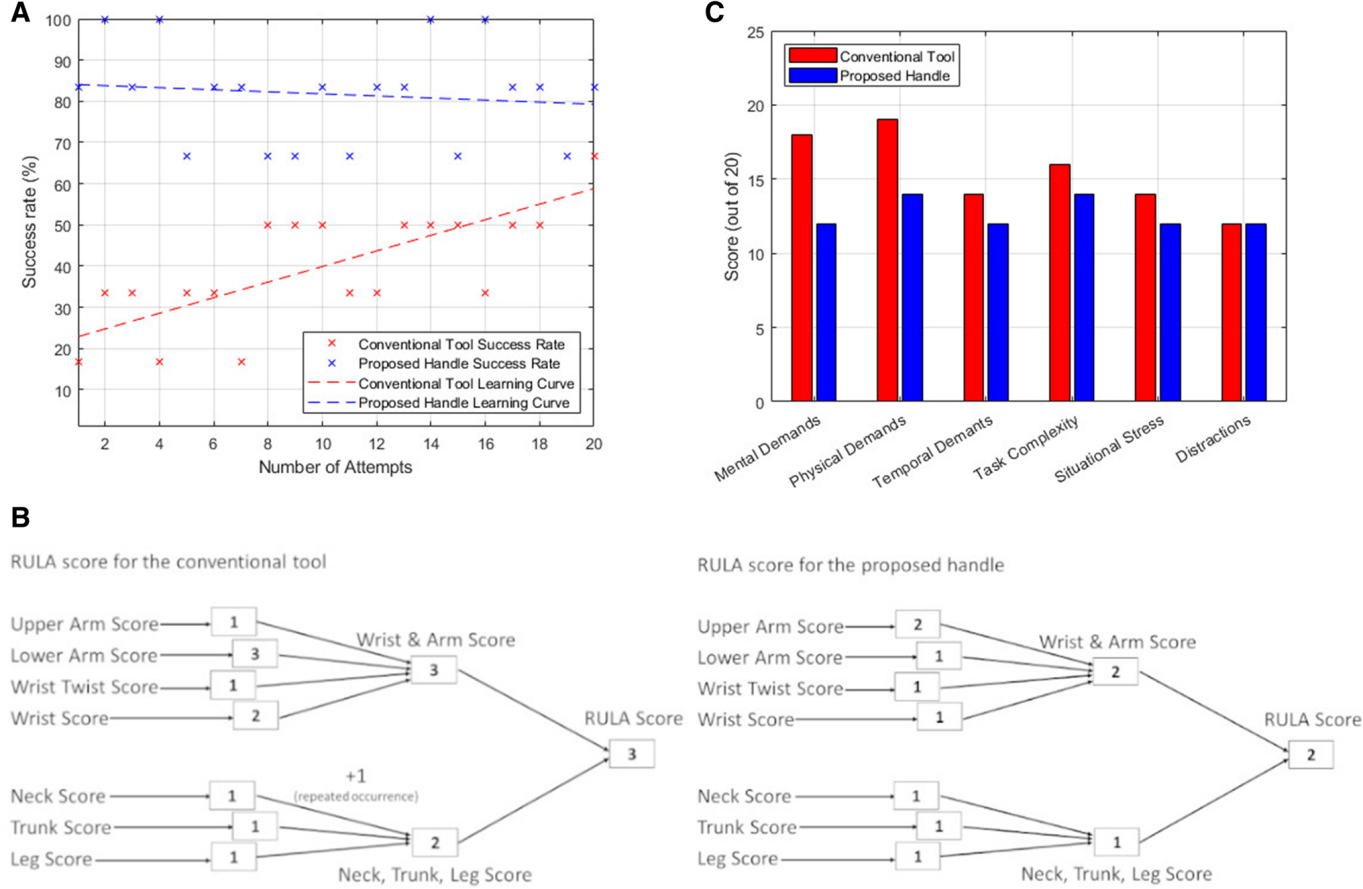
**a** Comparison graph of the success rates and learning curves of the two instruments, **b** The RULA score decision trees, and **c** the SURG-TLX questionnaire scores

In Fig. 7a, the graph compares the surgeon’s success rate, as previously defined, between the conventional tool and the proposed handle. In the same graph, we visualize the learning curve as the linear curve fitted to the function of the success rate over the number of attempts. As shown in [18], it is very difficult to define a generally accepted learning curve definition. In [31] and [7], however, 20 attempts were enough to evaluate it.

On the RULA measure scale, using the conventional tool the RULA score was 3 at the worst posture throughout the procedure, whereas with the proposed handle, the same RULA score was 2. A score of 3 falls under the ‘further investigation, change may be needed’ category, with a score of 2 falling under the ‘acceptable posture’ category. The RULA score decision trees are shown in Fig. 7b. Finally, the results of the SURG-TLX questionnaire are presented in Fig. 7c.

## Discussion

In this study, a novel intuitive handle prototype for a neurosurgical robotic instrument was presented. The handle is forearm-mounted and has a 4-DoF joystick that maps the surgeon’s wrist directly to the robot joints. To test for the efficacy and limitations of the handle, a preliminary comparative experiment between the suggested prototype and a standard neurosurgical tool was carried out.

### Principal findings

The suggested prototype outperformed the neurosurgical tool in terms of number of attempts until first completion, average success rate and completed tasks rate. In fact, the surgeon that tested the instruments was not able to complete the task using the conventional handle, even though they had extensive experience using such instruments. This was due to the fact that some pegs were placed outside the effective workspace of the standard tool, but within the normal workspace of neurosurgical procedures as mentioned in “Experimental methods” section.

It is self-evident that an articulated end effector would result in an expanded workspace when compared to a conventional tool, a trait that could be used by different input devices such as haptic controllers. However, in this study we are focusing on the implementation of a handheld tool that is mounted on the surgeon’s forearm, something that could theoretically lead to loss of dexterity. The proposed handle, however, still outperformed the conventional tool, and thus, the results of the study indicate that the slight loss in manipulability from constraining the forearm does not necessarily lead to loss of robotic dexterity.

The learning curves plotted in Fig. 7a showcase that when the surgeon was using the conventional tool, no plateau had been reached by the 20th attempt, whereas with the proposed handle, the learning curve is almost nonexistent with an immediate improvement in success rate. Although these findings are promising and could indicate toward fast tool adoption, further investigation with more user subjects will be conducted. This limitation in user subjects is especially telling in the case of the SURG-TLX questionnaire results, where although the improved evaluation could potentially prove encouraging, its small study size and subjective nature do not allow for concrete conclusions to be reached yet.

Finally, one of the goals set early on in the development of the handle prototype was the design of an ergonomic and comfortable to use handle. Based on the preliminary RULA scores, the worst posture adopted throughout the test when using the suggested handle was deemed acceptable.

### Limitations and future work

Despite the promising preliminary results for the proposed handle, there are some limitations that need addressing in future efforts. Of all the design criteria defined in “Design criteria” section, the adaptation for variable surgeons’ hand sizes and the potential incorporation of robotic assistance were not addressed in the current implementation. The adjustable straps and large joystick size can potentially cater to different hand sizes, whereas some handle components, such as the potentiometers, could be replaced by devices that offer force-feedback or gravity compensation. Another iteration of this forearm-mounted handle could replace the three-axis gimbal joystick with a standard two-axis joystick and switch. This, however, would decrease intuitiveness and exclude the roll DoF. Moreover, another implementation that could alleviate the surgeon of the added wrist strain could be a tele-operational platform.

When this handle prototype is incorporated in a final robotic instrument, it is certain that some redesign iterations will be required to address new electronics added and new robotic assistance that is yet to be implemented. Naturally, the weight of the design will also change, although not drastically since miniature motors and electronics will be used to control the miniature end effector. To validate the robotic prototype, a similar peg-transfer task could be deployed, only this time in a realistic physical scenario with real pegs and rings.

Regarding the simulation, the conventional tool’s poorer performance can be attributed in part to the fact that the experienced user subject has developed a preferred physical operating setup that did not necessarily match our broader experimental setup. Simultaneously, the fact that the surgeon used the conventional tool first could have contributed to the better performance of the proposed handle. However, we have indication to believe that this was not significant. The two simulated tasks differ substantially depending on the tool used because of the distinctively different manipulation means of the tools. The fact that the surgeon could achieve the task with the proposed handle after only two attempts, despite being unfamiliar with how it operates, could still suggest the absence of a learning curve beyond familiarity with the simulation environment.

Finally, despite the limitations of a single surgeon user, it helped to illustrate some of the potential of our proposed handle. The development of a user-facing neurosurgical robotic instrument, however, requires a larger user study where a group of medical trainees and experts test its handle prototype in random repetition patterns as to further investigate whether the familiarity with the setup affects the learning curve. Using the same simulated ‘peg-transfer’ task developed in this paper, the results and findings from a larger study can drive the evolution for the design and further evidence or verification of the improvement over the state of the art.
